# Integrated biodiesel and biopolymer production from *Nannochloropsis* biomass: a closed-loop biorefinery approach

**DOI:** 10.1039/d5ra02952j

**Published:** 2025-11-03

**Authors:** N. Nirmala, J. Arun, Sivasubramanian Palanisamy, R. S. Ernest Ravindran, Mohamed Abbas, Shaeen Kalathil, Md Zillur Rahman

**Affiliations:** a Centre for Waste Management, Sathyabama Institute of Science and Technology Jeppiaar Nagar Chennai Tamil Nadu 600119 India nirmala.amutha1189@gmail.com; b Centre of Excellence for Energy Research, Sathyabama Institute of Science and Technology Jeppiaar Nagar Chennai Tamil Nadu 600119 India; c Department of Mechanical Engineering, PTR College of Engineering & Technology Madurai – Tirumangalam Road Madurai Tamil Nadu 625008 India sivaresearch948@gmail.com; d Department of Electronics and Communication Engineering, Koneru Lakshmaiah Education Foundation Vaddeswaram Guntur Andhra Pradesh 522501 India; e Electrical Engineering Department, College of Engineering, King Khalid University Abha 61421 Saudi Arabia; f Department of Condensed Matter Physics, Saveetha School of Engineering, Saveetha Institute of Medical and Technical Sciences (SIMATS) Thandalam Chennai 602105 India; g Department of Electrical Engineering, College of Engineering, Princess Nourah bint Abdulrahman University P.O. Box 84428 Riyadh 11671 Saudi Arabia; h Department of Mechanical Engineering, Ahsanullah University of Science and Technology Dhaka 1208 Bangladesh md.zillur.rahman.phd@gmail.com

## Abstract

This study investigates the potential of utilizing deoiled algal biomass (DAB) derived from *Nannochloropsis* sp. after biodiesel production for biopolymer synthesis in a closed-loop system. The investigation demonstrates the feasibility of employing a biorefinery-based approach to produce both biodiesel and biopolymer, specifically polyhydroxybutyrate (PHB), from algae residues. Algal oil was extracted using the Bligh and Dyer method, with an optimal lipid yield achieved at 45 °C after 30 minutes, resulting in a lipid content of 29.41 wt%. Biodiesel conversion of the extracted oil was achieved with a 90.4 wt% efficiency using NaOH as a catalyst. The residual deoiled cake was further utilized for PHB production, achieving a maximum yield of 0.39 g PHB per g DOC (39% of DOC dry weight). The results highlight the potential of integrating biodiesel and biopolymer production into a single biorefinery process, promoting resource efficiency and sustainability. This approach highlights the viability of a circular bioeconomy for microalgal biorefinery systems, with minimum waste generation and maximum resource utilization.

## Abstract 2

A closed-loop biorefinery using *Nannochloropsis* sp. achieved 90.4% biodiesel conversion and 39% PHB yield from deoiled biomass, demonstrating sustainable dual production of biofuel and biopolymer for a circular bioeconomy.

## Graphical abstract

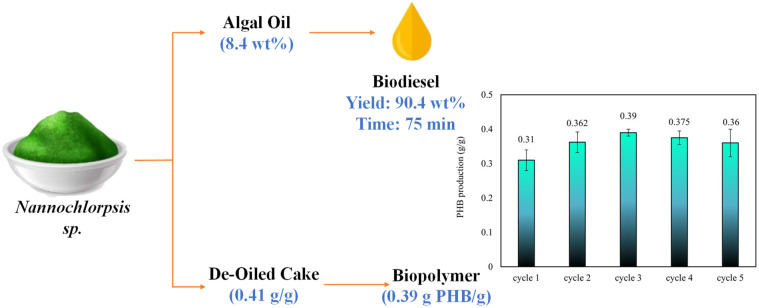

## 1. Introduction

Global warming, driven by the emissions of greenhouse gases, is one of the most pressing environmental challenges of the 21^st^ century. In this context, microalgae, small single-celled organisms, are emerging as a promising source for biodiesel production, offering an innovative renewable energy solution.^1^ Microalgae are capable of converting light, CO_2_, and nutrients into lipids and other organic molecules, which can be used to produce biodiesel. Through the assimilation of ambient CO_2_, microalgae store carbon in the form of lipids and carbohydrates, effectively generating carbon reserves.^2^ As a third-generation feedstock, microalgae outperform second-generation feedstocks such as lignocellulosic substrates (plant biomass derived from land), with microalgal biomass production being five to ten times faster. Unlike lignocellulosic feedstocks containing lignin, microalgae do not, making them a more economical choice for algal biorefineries, where the absence of lignin reduces the costs associated with ethanol production. However, on a commercial scale, microalgae cultivation remains economically challenging due to high capital and operational expenditures, as well as substantial energy requirements.^3^ Overcoming these economic constraints can be achieved by fully utilizing algal biomass and producing a diverse range of products to maximize the net energy output of algal bioprocesses.^4^

Microalgal cultivation offers numerous environmental and resource-based advantages.^5^ The high biomass yield of microalgae can be achieved in relatively small areas, providing a viable solution to space limitations.^6^ Furthermore, microalgae play a significant role in oxygen production, releasing over 70% of the environment's oxygen while absorbing approximately 1.8 pounds of CO_2_ for every pound of biomass produced.^7^ Although large-scale cultivation remains challenging, microalgae can be produced cost-effectively at smaller scales or under optimized conditions, requiring minimal maintenance, low operational inputs,^8,9^ and no need for complex machinery. Additionally, the utilization of wastewater or non-arable land further enhances the economic and environmental feasibility of microalgal biomass production.^6,10^ The simplicity of the cultivation system and the low costs associated with storing and transporting the product further enhance its economic viability.^9,10^

In recent years, advances in biomass valorization have led to a shift from single-product extraction toward integrated conversion pathways that combine catalytic, photochemical, and electrochemical upgrading with biological approaches to enhance overall carbon and energy yields.^11^ Photocatalytic and electrochemical strategies have been successfully employed to upcycle liquid biomass and model intermediates, demonstrating potential for decentralized or low-temperature fuel production routes that complement biological transesterification processes.^12,13^ These developments underscore the importance of coupling improved catalytic conversion techniques with efficient downstream processing to enhance the economic competitiveness of biofuels derived from non-conventional feedstocks, including algal oils.^14^

Continued progress in process intensification, catalyst design, and cost optimization is expected to bridge the gap between laboratory-scale and pilot-scale biodiesel production from alternative feedstocks. Studies on acid-base bifunctional catalysts and biomass-derived catalytic systems have reported enhanced conversion efficiencies and reduced environmental impacts.^15^ Moreover, life-cycle and techno-economic assessments emphasize the necessity of co-product valorization to offset production costs. In this context, integrating biodiesel production with the recovery of high-value co-products, such as polyhydroxybutyrate (PHB), from deoiled algal cake is considered a promising strategy to enhance the overall economic and environmental sustainability of algal biorefineries.^16,17^

The applications of microalgae span a range of industries, particularly in food, beverages, and pharmaceuticals.^18^ Microalgae have attracted considerable attention due to their high nutritional profile, which is rich in proteins, carbohydrates, lipids, and carotenoids, making them a valuable source of nutritional supplements and alternative proteins.^19,20^ Microalgal cells are also rich in essential vitamins, such as B12 and minerals, making them popular in powders, tablets, and capsules.^21,22^ The growing demand for plant-based proteins and dairy alternatives has further accelerated the use of microalgae in these sectors,^3,23,24^ with species such as *Spirulina* commonly being added to energy drinks and juices to improve their nutritional content.^25,26^

In addition to these applications, there is increasing interest in using microalgae for biopolymer synthesis.^9,20,27^ Among these, polyhydroxyalkanoates (PHAs), particularly polyhydroxybutyrate (PHB), have emerged as promising alternatives to conventional petrochemical-based plastics due to their biodegradability, biocompatibility, and favorable mechanical properties, which are comparable to those of synthetic polymers.^25,26^ The global PHA market has experienced rapid expansion in recent years, driven by increasingly stringent environmental regulations and rising consumer demand for sustainable packaging materials. Specifically, the PHB sector is projected to achieve substantial growth in the coming decades, reaching a multibillion-dollar valuation, supported by applications in medical devices, food packaging, and agriculture.^7^

At present, most PHB production relies on heterotrophic bacterial fermentation using sugar- or starch-rich substrates. These feedstocks compete with food sources and are associated with high substrate and processing costs, thereby constraining large-scale deployment.^8^ Moreover, downstream recovery processes are energy-intensive, and the requirement for sterile fermentation conditions further increases capital and operational expenditures, limiting the economic feasibility of PHB production.^26^

Microalgae-based PHB synthesis represents a sustainable and resource-efficient alternative to conventional approaches. Microalgae can grow autotrophically using sunlight and CO_2_, eliminating the need for sugar feedstocks while enabling cultivation on non-arable land or in wastewater, thus reducing resource competition and contributing to carbon capture.^6,8^ Under nutrient stress conditions, microalgae can accumulate intracellular PHB, which can subsequently be recovered from residual biomass following lipid extraction. Integrating such processes within a biorefinery framework enables maximal resource utilization and minimal waste generation.^3,5^ This dual valorization strategy, which involves producing biodiesel from lipids and PHB from deoiled biomass, offers a robust pathway toward economically viable and environmentally sustainable circular bioeconomy models.

Polyhydroxyalkanoates (PHA), a well-known biopolymer, can be synthesized from microalgae.^7^ These polymers are produced by accumulating neutral lipids in microalgal cells under specific environmental conditions.^8^ PHAs are microbial biopolyesters composed of hydroxyalkanoic acid monomers (commonly C3–C6). In optimized formulations (*e.g.*, copolymers, high molecular weight, and tailored crystallinity), their mechanical and thermal properties can approach those of conventional plastics, although issues such as brittleness and narrow processing windows may remain.^28,29^ PHAs can be categorized into three types based on their carbon chain length: short-chain PHAs (less than five carbon atoms), medium-chain PHAs (six to fourteen carbon atoms), and long-chain PHAs (more than fifteen carbon atoms).^30^ PHA production from microalgae can be achieved by cultivating the microalgae under stress conditions or through the thermo–mechanical polymerization of proteins extracted from microalgal biomass.^5,31^

While the first generation of biopolymer synthesis relies on renewable biomass and agricultural sources, it often involves high maintenance costs, substantial land use, and significant water consumption.^29,31^ The third-generation biopolymer production, utilizing microalgae, addresses these limitations by reducing operational costs, land requirements, and resource consumption.^29^ Microalgae cultivation can be conducted on a small scale with high yields, further minimizing costs, while also being capable of growing in wastewater, reducing resource costs, and eliminating the need for media sterilization.^32^ Additionally, using wastewater for microalgal cultivation promotes waste management and contributes to a circular economy, reducing carbon footprint and promoting environmental sustainability.^6,33^

This study aims to explore the processes involved in microalgae-based biodiesel production, including cultivation, harvesting, cell disruption, lipid extraction, transesterification, and the prospects and challenges associated with this production. Techniques for microalgal cell disruption, such as mechanical, chemical, and biological methods, are employed to extract lipids for biodiesel production.^34^ The by-product, “deoiled cake (DOC)” or “deoiled algal biomass”, remains after lipid extraction and holds significant potential for further valorization.^35^

The DOC contains abundant recyclable carbon compounds and sugars that can serve as precursors for producing biopolymers, bioethanol, volatile fatty acids, and hydrogen.^3^ While previous studies have primarily focused on synthesizing biopolymers from whole algal cells (Table 1), the present study investigates the potential of deoiled algal biomass as a feedstock for biopolymer production. Specifically, it explores the synthesis of biopolymers from deoiled *Nannochloropsis* sp. biomass following biodiesel extraction, advancing a closed-loop, zero-waste approach. This integrated valorization strategy couples biodiesel production with biopolymer synthesis, thereby enhancing the economic feasibility and environmental sustainability of algal biorefineries. The novelty of this work lies in the dual-purpose utilization of *Nannochloropsis* sp. for both biodiesel and biopolymer generation using residual biomass typically considered as waste. This approach maximizes biomass utilization, minimizes waste output, and reduces the overall environmental footprint of algal cultivation.

**Table 1:** PHB/PHA production from algal biomass

Algal species	Biopolymer type	PHB/PHA yield	Key findings	Study
Mixed marine microalgae	PHB	0.25 g g^−1^	PHB accumulation achieved in mixed cultures under nutrient stress	7
*Chlorella vulgaris*	PHA	32% DCW	PHA harvested in batch cultures during nutrient depletion	9
*Chlorella* sp	PHB	0.43 g g^−1^	High PHB yield obtained from whole cells *via* thermo–mechanical polymerization	26
*Scenedesmus* sp	PHB	0.30 g g^−1^	Sequential stress conditions enhanced PHB yield	5
Mixed microalgae	PHB	35% DCW	Two-step cultivation enabled PHA-based film production from whole-cell biomass	20
*Stigeoclonium* sp. B23	PHB	6.1 ± 0.07 g L^−1^	Highest PHB yield achieved with increased acetate and bicarbonate in nitrate-free medium	36
*Ulva* sp	PHA	3.79 g L^−1^	Acid pre-treatment improved PHB yield	37
*Cyanobacteria consortia*	PHB	28% DCW	Microbial consortia enriched through alternating growth and accumulation cycles	38
*Synechococcus* sp. MA19	PHB	55% DCW	Thermophilic strain produced PHB efficiently under phosphate-limited conditions	39
*Chlorella*	PHA	75.4% DCW	Defatted *Chlorella* biomass used as a carbon source for *Cupriavidus necator* cultivation	40

## 2. Materials and methods

### 2.1. Algal cultivation and harvesting

The algae *Nannochloropsis* sp. were obtained from NCL Pune, India. The culture was maintained under controlled conditions at 28 ± 2 °C and a pH of 7. The algal cultures were maintained under a 12 h light/12 h dark photoperiod using cool-white fluorescent lamps (Philips T8, 6500 K) at an intensity of 4500 ± 50 lux. Cultivation was conducted in 500 mL Erlenmeyer flasks for 20 days. Following growth, the biomass was harvested using Whatman No. 41 filter paper with a particle retention size of 20 μm. In a parallel approach, the microalgae *Nannochloropsis* sp. were also harvested by centrifugation of the grown culture medium at 4000 rpm for 10 minutes using a cooling centrifuge. The obtained biomass yield concentration was 2.9 g L^−1^. For comparison, a biomass yield of approximately 3.02 g L^−1^ was reported in previous studies using *Chlorella vulgaris*.^32^

### 2.2. Biomass characterization

The elemental composition of the biomass was analyzed using an elemental analyzer (PerkinElmer 2400 Series CHNS Analyzer). The algal biomass and its moisture content and ash content were measured following ASTM standards E871 and E1755.^41^ A thermogravimetric analyzer (Shimadzu TGA 50H) was employed to assess the thermo-gravimetric degradation of the biomass. In this experiment, 15 mg of biomass was heated at a rate of 5 °C per minute for 10 minutes until a final temperature was reached.^42^ The higher calorific value (HHV) of the biomass was determined using eqn (1).1
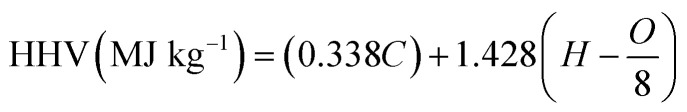
where *C* is the mass fraction of carbon in the biomass, *H* is the mass fraction of hydrogen, and *O* is the mass fraction of oxygen.

### 2.3. Extraction of algae oil from *Nannochloropsis sp*

Lipid extraction was performed following a modified Bligh and Dyer method.^48^ Approximately 1 g of freeze-dried algal biomass was homogenized with a mixture of methanol, chloroform, and water in a volumetric ratio of 2 : 1 : 0.8 (v/v/v). Initially, 20 mL of methanol was added to the biomass and stirred for 20 min to enhance solvent penetration. Subsequently, 10 mL of chloroform was added, and the mixture was stirred for an additional 5 min. Thereafter, 8 mL of distilled water was gradually added under continuous stirring to promote efficient cell disruption and lipid partitioning.^43^ The resulting suspension was centrifuged at 4000 rpm for 10 min at room temperature to facilitate phase separation. The lower organic phase containing lipids was carefully collected using a separating funnel. To maximize lipid recovery, the extraction process was repeated with the residual biomass. The combined chloroform extracts were concentrated using a rotary evaporator (RV 10, IKA, Germany) under reduced pressure at 45 °C to obtain crude algal oil.^44^ The maximum lipid yield reached 29.41 wt%, corresponding to a DOC yield of 0.42 g after 30 min of extraction.

### 2.4. Production of biodiesel from algae oil

After cultivation, the microalgae cells were centrifuged at 3500 rpm for 10 minutes. The resulting pellets were washed repeatedly with distilled water, discarding the supernatant after each wash. The pellets were then dried in a hot air oven at 70 °C until a constant weight was achieved. To a test tube containing 10 mg of dried biomass, 2 mL of a 2 : 1 (v/v) chloroform–methanol mixture was added.^45^ The test tubes were vigorously stirred for 20 minutes to ensure thorough mixing. Following this, 1 mL of methanol and 300 μL of concentrated H_2_SO_4_ were added, and the mixture was incubated at 100 °C for 20 minutes to promote transesterification.^29,30^ After allowing the tubes to cool to room temperature, 1 mL of distilled water was added. Phase separation was facilitated by centrifugation (K241 Centurion Scientific, UK) at 3500 rpm for 5 minutes. The lower phase, containing the methyl esters, was carefully collected using a syringe and subsequently filtered through a 0.45 μm ANOW lab syringe filter to obtain the FAME (fatty acid methyl ester) and a chloroform solution.^46^ The conversion efficiency was calculated using the following equation.2
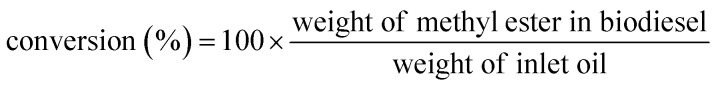


### 2.5. Production of biopolymer using the obtained DOC from *Nannochloropsis sp*

The biopolymer synthesis (*i.e.*, PHA) was carried out using the carbon source derived from the DOC. Initially, sugars obtained from DOC were utilized to enrich a PHA-producing microbial culture. The microbial culture was supplemented with nutrients in a ratio of 100 : 8 : 1, which included sodium nitrate (NaNO_3_) and dipotassium phosphate (K_2_HPO_4_).^21^ The first phase, termed the “feast phase” (Stage 1), involved providing the microbial culture with DOC sugars as the primary substrate for microbial enrichment. Subsequently, nutrient levels were reduced during the “famine phase” (Stage 2), and the culture was fed exclusively with DOC sugars to stimulate biopolymer accumulation.

For the fermentation process, oxygen was supplied externally *via* an air pump to 100 mL borosilicate bottles used in the experiments.^47^ The sequencing batch reactor mode was employed with a 48-hour half-life for reactor operation. The three-step optimal condition approach described in the study was used for the effective extraction of the biopolymer.^48^ The bacterial pellets were harvested and subjected to two washing steps at 4000 rpm for 10 minutes at 28 ± 0.2 °C using a phosphate buffer (50 mM). Then, an equal volume of sodium hypochlorite and chloroform was added to the washed pellets. The resulting mixture was incubated for three hours at 120 rpm and 32 ± 0.5 °C in a shaking incubator. After incubation, the mixture was centrifuged at 6000 rpm for 10 minutes at 32 ± 0.2 °C. Post-centrifugation, the three distinct chloroform layers were separated and combined with ice-cold methanol. The polymer began to precipitate as methanol was added to chloroform. The samples were then repeatedly cleaned with methanol and stored at 4 °C for 12 hours. The polymer was then weighed and dried for 60 minutes at 60 °C. Up to five recycling cycles were performed, and the yield of biopolymer production was calculated. The PHB yield (*Y*_PHB_) was calculated as the mass of PHB produced per mass of DOC on a dry weight basis using eqn (3). A schematic diagram illustrating the mechanism of biopolymer formation is shown in Fig. 1.3
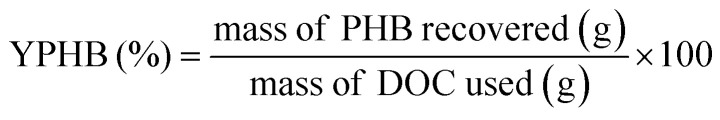


**Fig. 1:**
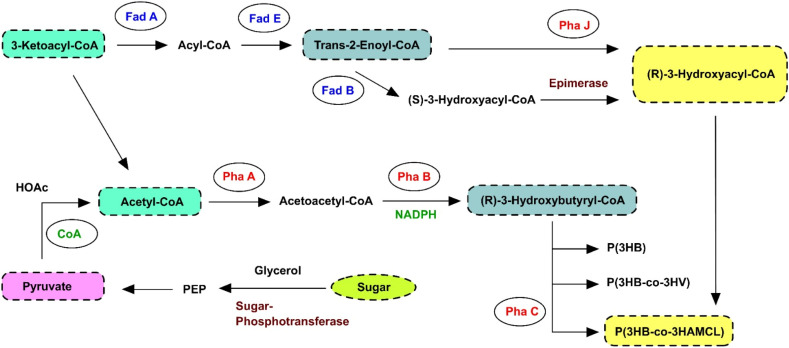
Schematic diagram of the mechanism of biopolymer formation.

### 2.6. Characterization of biodiesel

The physical and chemical properties of the biodiesel were evaluated in accordance with ASTM standards. These included density (measured using the hydrometer method, ASTM D1298), acid value (ASTM D664), specific gravity, viscosity (determined with the Universal Lab Product, ASTM D445), flash point (measured using the Deep Vision Instrument, ASTM D130), and copper strip corrosion, as outlined in ref. 49 and 50.

### 2.7. Characterization of biopolymer from DOC

Biochemical techniques were employed to quantify the sugar content in the DOC before and after the biopolymer synthesis. To assess its physicochemical properties, the biopolymer was subjected to TGA using a Shimadzu TGA-50H instrument, and structural characterization was conducted *via* Fourier-transform infrared spectroscopy (FT-IR) using a PerkinElmer FTIRC-100566 (Japan). Thermal analysis was performed under a nitrogen atmosphere with a controlled heating rate of 5 °C min^−1^, spanning a temperature range of 100 to 800 °C.

## 3. Results and discussion

### 3.1. Effect of temperature on lipid yield

**Fig. 2:**
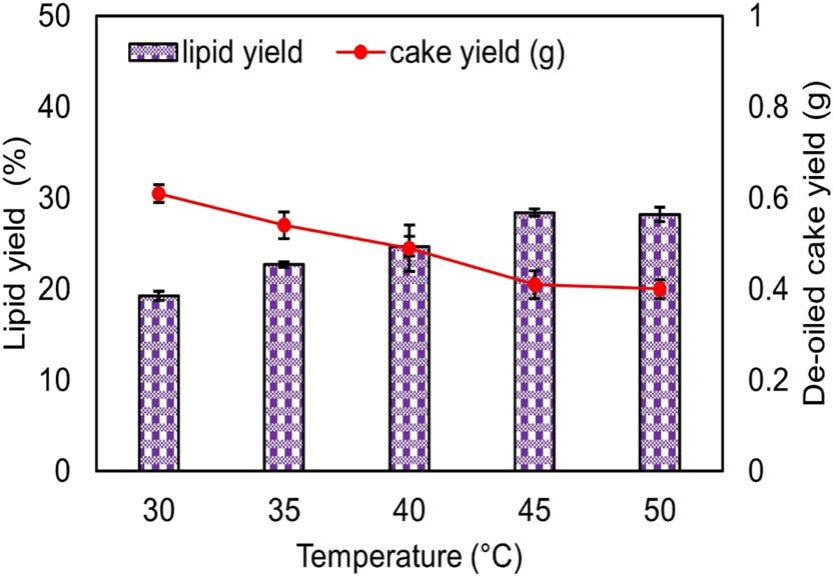
Effect of temperature on lipid yield.

Fig. 2 illustrates the relationship between temperature (°C), lipid yield (%), and cake yield (g), with the right *Y*-axis representing the DOC yield (g) (the absolute mass of the deoiled biomass recovered after extraction). As the extraction temperature increased, the lipid yield also increased, reaching a maximum of 29.41 wt% at 45 °C. A further rise in temperature to 50 °C resulted in a marginal change, with a lipid yield of 27.8 wt% compared to 26.4 wt% at 40 °C.^51^ The slight improvement in yield at elevated temperatures can be attributed to enhanced solvent diffusivity and reduced viscosity, which facilitate better penetration of the solvent mixture into the algal cell matrix and improve the dissolution of intracellular lipids. However, excessively high temperatures may induce thermal degradation of sensitive lipid fractions, thereby reducing the overall yield. Similar temperature-dependent trends in lipid extraction have been reported in a previous study.^32^

### 3.2. Effect of time on lipid yield

**Fig. 3:**
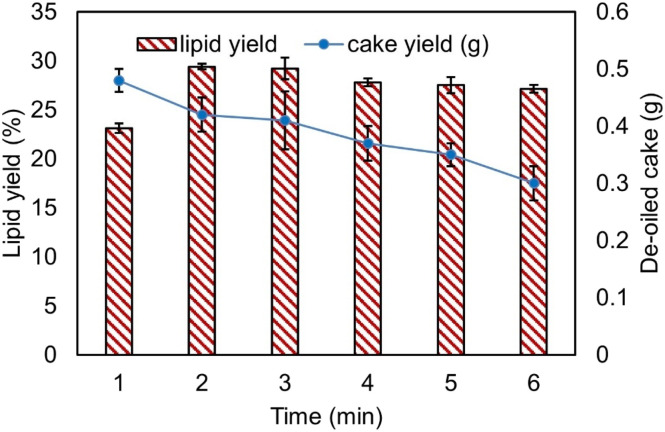
Effect of time on lipid yield.

Fig. 3 shows the relationship between extraction time (in minutes), lipid yield (%), and DOC yield (%). Lipid yield increases as the extraction time extends from one to three minutes, reaching a maximum of 30% at three minutes. After this point, lipid yield gradually declines between four and six minutes. This trend suggests that extending the extraction time beyond three minutes is unnecessary for optimal lipid recovery, potentially leading to degradation. Additionally, the yield of DOC steadily decreases from one to six minutes, indicating that longer extraction times result in more efficient lipid extraction and less residual oil in the cake. It can be inferred that an extraction time of approximately three minutes is optimal for achieving maximum lipid yield. For comparison, the Bligh and Dyer extraction method applied to *Scenedesmus* sp. resulted in a lipid yield of 14.5 ± 0.5% after two hours, highlighting the differences in yield based on extraction time.^29^

### 3.3. Effect of catalyst load and time on conversion of lipid into biodiesel

**Fig. 4:**
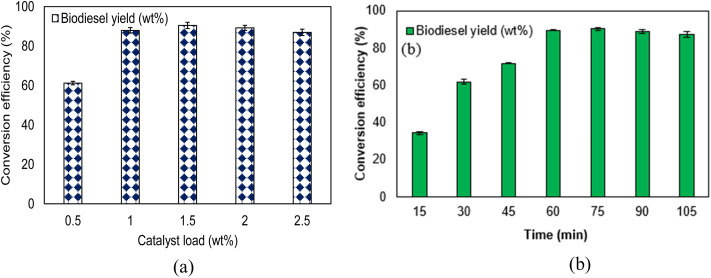
Effect of (a) catalyst load and (b) time on biodiesel production.

Fig. 4(a) depicts the influence of NaOH catalyst loading and reaction time on the conversion efficiency of algal oil to biodiesel. Each data point represents the mean of three independent experiments, and the error bars denote the corresponding standard deviations, reflecting the reproducibility of the results. As the catalyst loading increased from 0.5 g to 1.5 g, the biodiesel yield increased steadily, reaching a maximum of 90.4 wt% at 1.5 g NaOH. However, further increases in catalyst loading beyond this level resulted in a decline in yield, decreasing to 84.6 wt% at 2.5 g. This reduction is attributed to saponification reactions initiated by the excess strong base, which convert free fatty acids into soap rather than methyl esters.^2,29^ The soap formation increases the viscosity of the reaction medium and stabilizes emulsions, thereby hindering phase separation and preventing the loss of methyl esters in the soap phase.^36,37^ The effect of reaction time on conversion yield was also examined by varying the reaction duration from 0 to 105 minutes while maintaining a constant NaOH catalyst load, as shown in Fig. 4(b). The results indicate that the conversion yield increased with time, reaching a maximum of 90.4 wt% at 75 minutes. Beyond this point, a slight decline in yield was observed, with the value decreasing to 88.6 wt% at 90 minutes. The reduction in yield at extended reaction times may be due to the reversible nature of the transesterification reaction and secondary hydrolysis of the produced esters.

### 3.4. Properties of DOC and algal biodiesel

The physical and chemical characteristics of the produced biodiesel, including density, acid value, specific gravity, viscosity, flash point, moisture content, and copper strip corrosion, were determined following ASTM standard procedures. The biodiesel exhibited a density of 891 kg m^−3^, falling within the ASTM and BIS (Bureau of Indian Standards) standards range of 860 to 900 kg m^−3^. The specific viscosity measured 0.872, consistent with the ASTM and BIS standards, which stipulate values between 0.86 and 0.9. The acid value of the biodiesel was 0.31, which is well below the maximum allowable limit of 0.5 as per both ASTM and BIS standards. The viscosity was recorded at 3.91 cSt, within the acceptable range of 1.9 to 6 cSt according to ASTM and 2.5 to 6 cSt as per BIS standards.^52^ The flash point was determined to be 142 °C, which is higher than the minimum threshold of 120 °C, and falls within the ASTM and BIS range of 100 to 170 °C. The moisture content was 0.091%. The copper strip corrosion test showed a moderate tarnish (1a) after three hours of exposure at 60 °C. Overall, the biodiesel properties align with the parameters set by the ASTM and BIS standards.^53^ Biochemical analyses of the DOC revealed the following measurements: glucose, 0.51 ± 0.2; soluble sugar, 4.4 ± 0.2; xylose, 0.05 ± 0.1; arabinose, 0.07 ± 0.4; and pH, 4.4 ± 0.2.

### 3.5. PHB yield from DOC

**Fig. 5:**
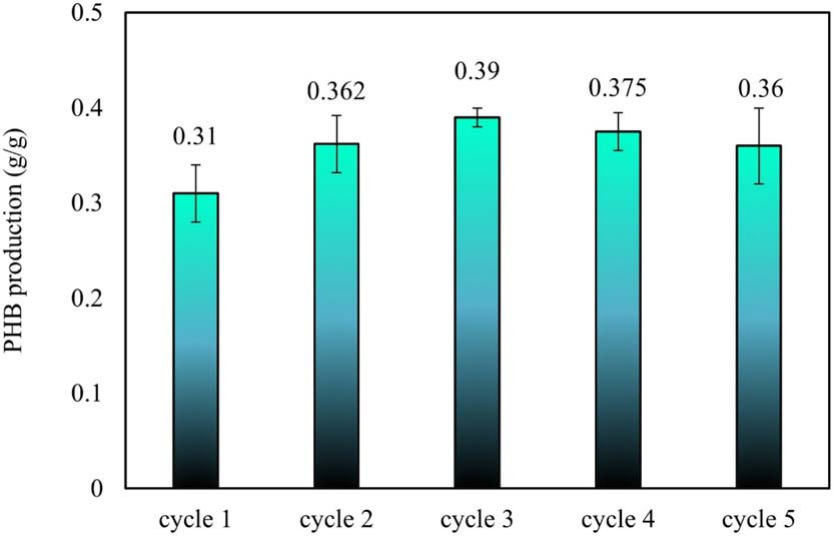
PHB yield from DOC.

The maximum PHB yield was approximately 0.39 g PHB per g DOC, corresponding to 39% of the DOC dry weight during Cycle III, as shown in Fig. 5. This peak in PHB production could be attributed to a potential depletion of the DOC nutrient supplement, which may have contributed to the increased accumulation of PHB. In contrast, the PHB yield in Cycle I was relatively low at 0.31 g PHB per g DOC. However, as the cycles were repeated, the yield progressively increased, reaching its highest point in Cycle V with a yield of 0.36 g PHB per g DOC. A similar trend was reported by Rajpoot *et al.*^14^

### 3.6. FTIR analysis

**Fig. 6:**
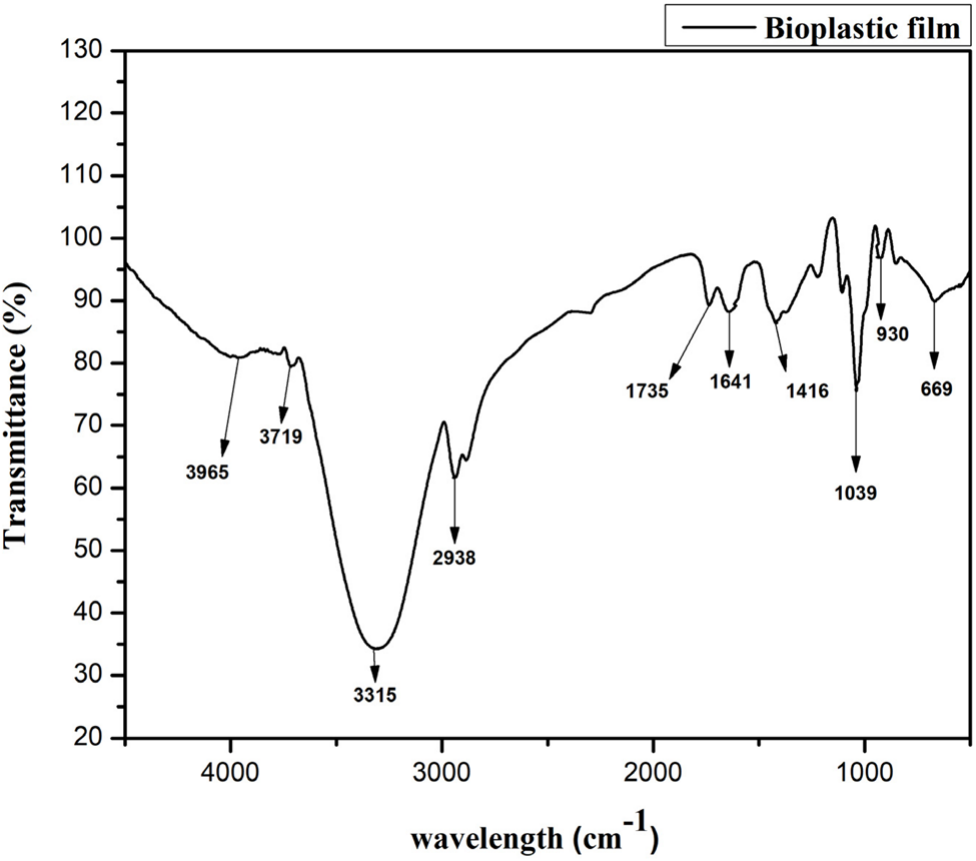
FTIR spectra of bioplastic film.

Fig. 6 displays the FTIR spectra of the bioplastic film, offering crucial insights into its chemical composition. Clear absorption peaks correspond to significant functional groups. A broad peak at 3315 cm^−1^, due to O–H stretching, indicates the presence of hydroxyl groups from hydrophilic substances, such as cellulose or starch.^3^ A weaker peak at 3719 cm^−1^ suggests the presence of free hydroxyl groups, likely originating from alcohols or unbound water.^16^ The peak at 2938 cm^−1^, characteristic of C–H stretching, corresponds to aliphatic hydrocarbons in the polymer chains.^34^ In bioplastics such as PLA, ester groups show C=O stretching, evident in the strong absorption at 1735 cm^−1^.^15^ The band at 1641 cm^−1^, typically associated with moisture or hydrogen bonding, corresponds to O–H bending.^11,54^ Additionally, the peak at 1039 cm^−1^, attributed to C–O stretching, suggests the presence of polysaccharides or plasticizers, while the absorption at 1416 cm^−1^ indicates C–H bending vibrations.^55^ Smaller peaks at 669 cm^−1^ and 930 cm^−1^ are associated with out-of-plane bending vibrations or crystalline structures.^56^ Overall, FTIR analysis highlights the presence of key functional groups—hydroxyl (O–H), carbonyl (C=O), and aliphatic (C–H)—which confirm the composite nature of the bioplastic film. The broad peak at 3315 cm^−1^ for O–H stretching and the peak at 1735 cm^−1^ for C=O stretching indicate the presence of hydrophilic components such as cellulose or starch and ester groups typical of PLA, suggesting potential interactions with moisture and plasticizers.

### 3.7. Thermal stability analysis

**Fig. 7:**
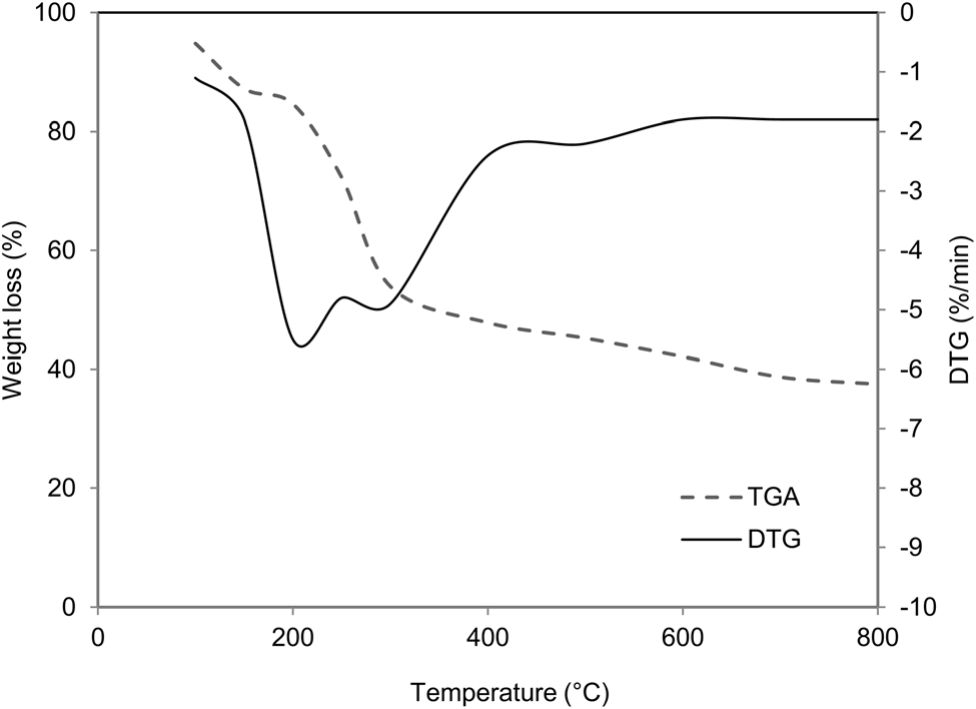
TGA analysis of bioplastic film.

The thermal degradation behavior of the algal-based biofilms is displayed in Fig. 7. The degradation was analyzed over a temperature range of 0 to 800 °C. Distinct weight-loss stages can be observed across different temperature intervals. The first stage, observed between 100 and 200 °C, showed a weight loss of approximately 10.2%, primarily attributed to the evaporation of physically adsorbed moisture and low-molecular-weight volatile compounds. The second major degradation phase occurred between 210 and 300 °C, corresponding to a weight loss of around 30.7%. The DTG curve exhibited a prominent peak at approximately 245 °C, representing the temperature at which the maximum degradation rate occurred. This stage is associated with the thermal decomposition of PHB and polysaccharides, which arises through random chain scission of ester linkages, releasing monomers such as crotonic acid and oligomers.^57,58^ The third stage, extending from 310 to 800 °C, accounted for an additional 10–12% weight loss. This slow degradation is attributed to the breakdown of residual carbonaceous and protein-derived components, as well as the oxidation of thermally stable aromatic or cross-linked residues. Beyond 700 °C, the mass loss stabilized, indicating minimal inorganic or ash content and confirming the primarily organic nature of the biofilm matrix. The final residual mass is ∼38% at 800 °C.

Overall, the algal-based PHB biofilms exhibited major degradation at approximately 245 °C, consistent with the typically reported maximum degradation rate temperatures for PHB-based composites (240–270 °C). The relatively high degradation onset and stable residue formation demonstrate good thermal stability, suggesting that these bioplastics are suitable for low- to moderate-temperature applications, such as biodegradable packaging, agricultural films, and eco-friendly composite materials.

### 3.8. Sugar removal

Table 2 presents the sugar content of DOC before its use as a substrate for biopolymer synthesis. In this study, the percentage of sugar removal in the substrate was measured at various reaction times during the PHB production. The sugar removal percentage increases with longer reaction times. Initially, the concentrations of glucose, soluble sugars, xylose, and arabinose in the DOC were 0.51 ± 0.2, 4.4 ± 0.2, 0.05 ± 0.1, and 0.07 ± 0.4 g per g DOC, respectively. After a reaction time of 120 hours, the levels of these sugars decreased by 50, 19, 68, and 69%, respectively, indicating that the available sugars were effectively utilized in PHB synthesis. Similarly, a maximum PHB production yield of approximately 0.43 ± 0.20 g PHB per g dry cell weight was achieved using deoiled algal biomass for biopolymer production in a study.^21^ Two other studies^29,59^ also reported a PHB production yield of 0.43 ± 0.2 g, with a substrate removal rate of 76.17%.

**Table 2:** Percentage removal of soluble sugar content in the DOC

Time (h)	Glucose	Soluble sugars	Xylose	Arabinose
12	24 ± 0.05	13 ± 0.03	37 ± 0.05	44 ± 0.08
24	31 ± 0.02	17 ± 0.05	43 ± 0.01	48 ± 0.01
36	37 ± 0.01	19 ± 0.07	48 ± 0.09	52 ± 0.03
48	41 ± 0.09	20 ± 0.01	53 ± 0.07	56 ± 0.04
60	43 ± 0.05	22 ± 0.09	57 ± 0.06	63 ± 0.07
72	45 ± 0.03	23 ± 0.03	60 ± 0.05	65 ± 0.05
84	49 ± 0.01	21 ± 0.01	63 ± 0.03	66 ± 0.06
96	53 ± 0.07	21 ± 0.04	61 ± 0.02	68 ± 0.04
108	51 ± 0.02	20 ± 0.06	60 ± 0.06	70 ± 0.01
120	50 ± 0.04	19 ± 0.09	61 ± 0.01	68 ± 0.03

## 4. Conclusion

This study successfully demonstrated the integration of biodiesel production and biopolymer synthesis using DAB derived from *Nannochloropsis* sp., highlighting the potential of a closed-loop biorefinery approach that maximizes resource utilization and minimizes waste generation. Optimal conditions for lipid extraction were identified at 45 °C and a 30-minute extraction time, achieving a lipid yield of 29.41%. The biodiesel production process reached a high conversion efficiency of 90.4% using NaOH as a catalyst, supporting the feasibility of large-scale biodiesel production. Additionally, the study achieved a maximum PHB yield of 0.39% (PHB per g DOC), demonstrating the potential of DAB for biopolymer synthesis and contributing to the growing field of sustainable bioplastics. This integrated approach promotes a circular bioeconomy by converting algal biomass into biodiesel and biopolymers, offering significant environmental and economic advantages. Future research should focus on optimizing PHB yields, exploring more efficient microbial strains, and scaling the process for industrial applications, which could help facilitate the production of commercially viable and environmentally sustainable biofuels and biopolymers.

## Author contributions

N. N.: writing – original draft, conceptualization, supervision; J. A.: writing – original draft, methodology, project administration; S. P.: writing – original draft, investigation, resources; R. S. E. R.: writing – review and editing, data curation, resources; M. A.: writing – review and editing, funding acquisition; S. K.: writing – review and editing, data curation, funding acquisition; M. Z. R.: writing – original draft, supervision, writing – review and editing.

## Conflicts of interest

The authors declare that they have no known competing financial interests or personal relationships that could have appeared to influence the work reported in this paper.

## Data Availability

All supporting data are available within the article.
